# Costs and cost-effectiveness of robotic-assisted surgery in South Korea: a systematic review and meta-analysis

**DOI:** 10.3389/fpubh.2025.1683482

**Published:** 2025-10-17

**Authors:** Young Eun Hong, Hana Shim, Minkyung Shin

**Affiliations:** Health Economics and Outcomes Research, Intuitive Surgical Korea, Seoul, Republic of Korea

**Keywords:** robotic assisted surgery, South Korea, health economics, systematic review, meta analysis

## Abstract

**Introduction:**

Robot-assisted surgery (RAS) has rapidly expanded across multiple surgical specialties since being introduced in South Korea. RAS has been proven clinically safe and effective, but its economic implications have not been thoroughly investigated. As public reimbursement for RAS is increasingly adopted by Asian countries such as Japan and Taiwan, the economic implications of RAS have become a critical factor in influencing reimbursement decisions in Korea.

**Method:**

We conducted a systematic review and meta-analysis of studies reporting cost and cost-effectiveness of RAS in Korea, published between 2007 and March 2025. Studies were searched using three databases: PubMed, EMBASE, and Scopus. Two authors independently performed literature screening, data extraction and risk of bias assessment using ROBINS-I and RoB. Outcomes were analyzed through meta-analysis with RevMan.

**Results:**

A total of 24 were included, comprising two randomized controlled trials (RCT) and 22 observational studies. Most studies were conducted at single institutions. Colorectal surgery was the most frequently studied procedure. For RAS, total hospitalization costs ranged between $6,762 and $20,206, while laparoscopic or endoscopic surgery spanned from $3,038 to $11,933, and open surgery costs ranged from $1,706 to $9,009. The meta-analysis demonstrated that the mean difference in total hospitalization cost between RAS and laparoscopic surgery was $3,279 (95% CI: $2,414 to $4,145; I^2^:95%).

**Conclusion:**

This is the first comprehensive systematic review and meta-analysis specifically assessing the economic implications of RAS in Korea. Our findings indicate that RAS is more costly than other surgical modalities at the time of surgery. However, the current review identified a lack of evidence on post-discharge costs, and no comprehensive cost-effectiveness or cost-utility analyses have been conducted in Korea. Future studies are encouraging to explore the long-term costs across different modalities.

## 1 Introduction

Robotic-assisted surgery (RAS) has expanded across various surgical specialties in South Korea after the first system was installed in 2005 (1, 2). This significant growth has been driven by the technical superiority of RAS which enables greater surgical precision, particularly in anatomical areas where conventional laparoscopic surgery poses significant challenges (3, 4). Increasing demand from patients, surgeons, and hospitals for innovative surgical care (5, 6), along with comparable or superior post-operative clinical outcomes (7, 8), has further accelerated RAS adoption.

During the early adoption phase, RAS was predominantly used in prostatectomy, followed by partial nephrectomy, pyeloplasty, and radical cystectomy (9). After that, the adoption of RAS has significantly expanded into other surgical specialties such as gynecology, thoracic, and general surgery. In gynecology, robotic- assisted hysterectomy accounted for 43% of all hysterectomies in 2021 (10). In colorectal surgery, RAS adoption has significantly grown, especially in complex procedures such as low anterior resection (LAR) (11). Thoracic surgeries, including lobectomy and thymectomy, have shown rapid adoption of robotic techniques in recent years (12).

As RAS utilization continues to rise, the economic implications of RAS are gaining more attention. The economic value of RAS, particularly in relation to potential savings in overall healthcare expenditure, has become a key consideration in public reimbursement decisions, especially in resource-constrained healthcare systems. In Asia, RAS is publicly reimbursed in Japan, Taiwan and Shanghai (China), with coverage extending to various surgical specialties (13–15). In South Korea, reimbursement for RAS has also been reviewed. The National Evidence-based Healthcare Collaborating Agency (NECA) conducted health technology assessments (HTAs) on RAS in 2011, 2014, 2015 and 2023 respectively (16). While these HTA reports confirmed the clinical safety and effectiveness of RAS in various procedures, cost-effectiveness evidence remains limited.

In this study, we conducted a systematic review of cost and cost-effectiveness studies on RAS in Korea, covering publications from 2007 to early 2025. The objective is to provide relevant stakeholders with updated RAS economic evidence to highlight key knowledge gaps, and identify priority areas for future efforts, including real world data collection, methodological improvements, and policy engagements.

## 2 Method and materials

### 2.1 Searching strategy

We searched PubMed, Embase, and Scopus to identify cost or cost-effectiveness literature of RAS in Korea. No Korean domestic databases (e.g., KoreaMed, KISS) were searched, as our review aimed to focus on studies indexed in internationally recognized databases to ensure comparability, accessibility, and reproducibility for global readership. The search period spanned from January 1, 2007, to May 8, 2025. Although RAS became available in Korea in 2005, literature databases only provide search services starting from 2007. The search terms we applied for the three databases were: “cost^*^ OR economic^*^ OR financial^*^ OR pric^*^ OR charge^*^ OR billing^*^) AND (Korea) AND (“robot surgery” OR “robot-assisted^*^” OR “robotic surgery” OR “robotic-assisted^*^”)” (Supplementary 1). The PICOS framework (Population, Intervention, Comparators, Outcomes, and Study Design) was used to guide the study selection criteria. The population included patients with either benign or malignant tumors. The intervention of interest was RAS. Comparators included open surgery, laparoscopic surgery, endoscopic surgery, or video-assisted thoracoscopic surgery (VATS). The outcomes focused on economic measures, such as total hospitalization cost, operation cost, government or patient payments. Eligible study designs included observational studies, cohort studies, and randomized controlled trials.

### 2.2 Study selection

Two researchers independently reviewed the literature and extracted data. Any inconsistencies during the study review were discussed by the researchers. Exclusion criteria were non-human studies, procedures not involving soft-tissue RAS, studies conducted based on datasets outside of South Korea, non-original articles, and non-English publications. Extracted data included the author(s), year of publication, study population, study design, study site, study period, cost components, and economic outcomes. The review was conducted in accordance with Preferred Reporting Items for Systematic Reviews and Meta-Analyses (PRISMA) guidelines (17) (Supplementary 1).

### 2.3 Risk of bias assessment

The risk of bias in randomized clinical trials (RCTs) was evaluated using the Cochrane Risk of Bias tool (RoB-2), while observational studies were assessed using the Risk of Bias In Nonrandomized Studies of Interventions (ROBINS-I) tool (18, 19).

### 2.4 Statistical analysis

For the meta-analysis comparing RAS with laparoscopic or endoscopic surgery, we extracted the following economic outcomes to generate forest plots with pooled estimates: total hospitalization costs (from admission to discharge), operation costs, patient out-of-pocket (OOP) costs, and government payments covered by the National Health Insurance Service (NHIS). We extracted mean, standard deviations (SDs) and sample sizes from the literature for data synthesis. For studies reporting median and interquartile range (IQR), we estimated the mean and SD using the method suggested by Luo et al. (20) and Wan et al. (21).

When comparing economic outcomes, all currencies were standardized to U.S. dollars (USD). While most studies reported costs directly in USD, some reported costs in Korean Won (KRW) or Euros (EUR). In those cases, the average exchange rate during the observation period of each study was applied to convert costs into USD. Inflation adjustments were not applied because most studies did not report the reference year for cost valuation, thereby preventing consistent adjustment.

Effect sizes were calculated as mean differences between RAS and laparoscopic groups. Meta-analyses were conducted using a random-effects model to account for the heterogeneity among studies. The Restricted Maximum-Likelihood (REML) random-effects model was used to account for heterogeneity. Heterogeneity was quantified by I^2^ statistic. The Hartung-Knapp-Sidik-Jonkman (HKSJ) method was used to estimate 95% confidence intervals. Forest plots were used to visually display the results of individual studies and the synthesized estimates. The analysis was performed with Review Manager (RevMan). To assess potential publication bias, funnel plots were generated, and Egger's regression test was conducted. R (4.3.1; Vienna, Austria) was used to assess the publication bias.

Additionally, to explore potential sources of heterogeneity in cost estimates, we conducted sensitivity analyses stratified by indication (benign vs. malignant) and by specialty (colorectal vs. non-colorectal). These analyses were performed using the same random-effects approach as the primary analysis, and results are presented in Supplementary 3.

## 3 Results

### 3.1 Study screening

**Figure 1:**
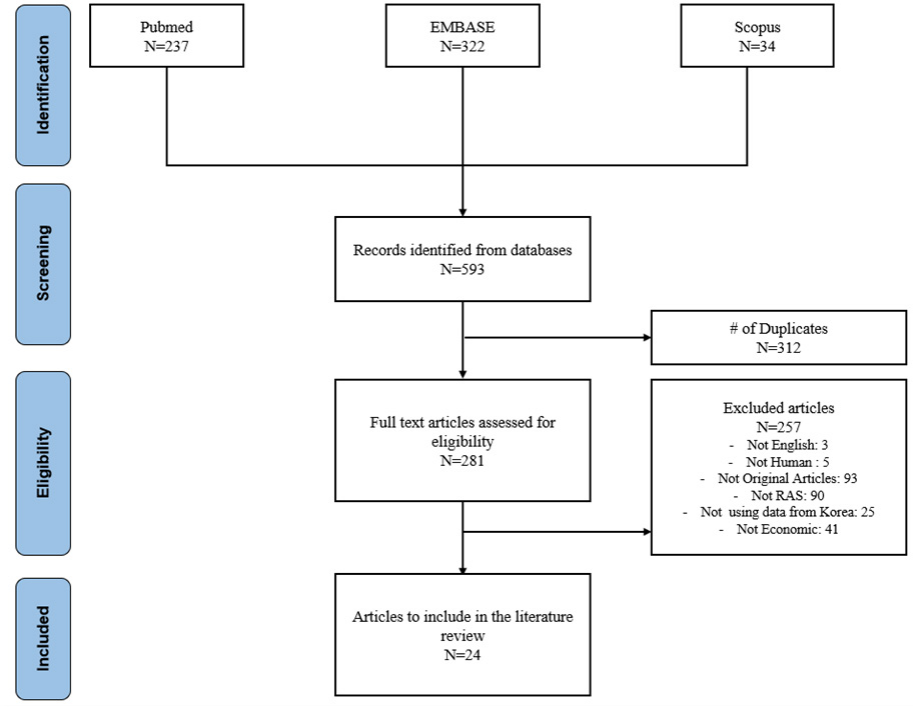
PRISMA Flowchart.

A total of 593 publications were identified in the initial search: 237 from Pubmed, 322 from Embase and 34 from Scopus. After removing 312 duplicates, 281 publications remained for title and abstract review. Of these, 257 articles were excluded: 5 studies were not written in English, 5 studies did not involve human subjects or soft tissue RAS, 93 were not original articles, 90 were not related to RAS, 25 studies were not conducted using Korea data, and 41 studies did not include economic outcomes. In the full manuscript review, 24 publications were included. The literature selection process is summarized in Figure 1.

### 3.2 Characteristics of selected literature

**Figure 2:**
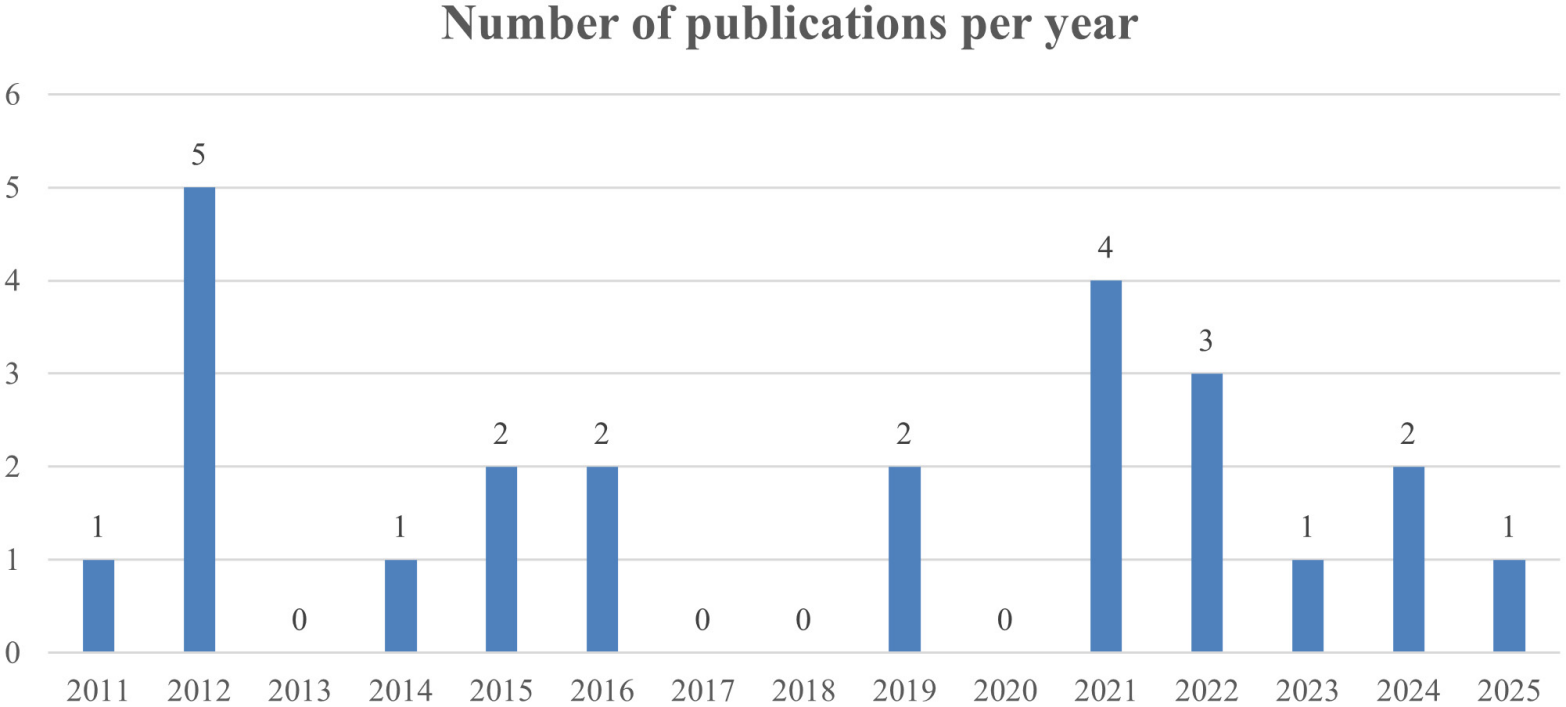
Number of publications per year.

Of the 24 studies selected, all were published from 2011 onward. The annual number of publications varied over time, without clear upward or downward trends. The highest number of publications occurred in 2012 (*N* = 5), followed by 2021(*N* = 5). On average, 1.26 RAS economic studies were published per year. No eligible studies were published in 2013, 2017, 2018, 2020 (Figure 2) Notably, no full economic evaluations, such as cost-effectiveness or cost-utility analyses using decision trees or Markov models, were identified. The majority of the studies were observational studies: 19 retrospective cohort studies and 3 prospective studies. Two studies were RCT. Regarding study methodology, 5 studies used statistical adjustment methods including propensity score matching (PSM) and 19 studies directly compared the outcomes without adjustment. In terms of institutional settings. Twenty-one studies were conducted at single institutions. Two were multi-institution studies. One study utilized a NHIS database. Yonsei University Severance Hospital was the most published site. Among the single institutions, 14 studies were conducted at upper general hospitals, a tertiary referral hospital, and seven at general hospitals (Table 1).

**Table 1:** Characteristics of selected studies.

**Included studies**	**Procedure**	**Population**	**Data collection year**	**Time frame**	**Healthcare cost included**	**Total Cost (mean)**			**Operation Cost (mean)**			**Patient Payment (mean)**			**Government Payment (mean)**		
						**RAS**	**LAP/END/VATS**	**Open**	**RAS**	**LAP/END/VATS**	**Open**	**RAS**	**LAP/END/VATS**	**Open**	**RAS**	**LAP/END/VATS**	**Open**
Kang et al. (22)	Pancreatectomy	45 (25 LAP, 20 RAS)	March 2006 to July 2010	Hospitalization	Total cost, operation cost	8,305	3,862	–	5,753	2,222	–	–	–	–	–	–	–
Park et al. (45)	Radical Nephrectomy	80 (20 LAP, 20 RAS, 20 OPEN, 20 VAMS)	January 2008 to December 2010	Hospitalization	Total cost, procedure and operation, anesthesia, laboratory, medical supplies	6,762	3,038	1,760	5,968	533	–	6,004	475	–	758	2,562	–
Park et al. (23)	Distal gastrectomy	150 (120 LAP, 30 RAS)	March 2010 to May 2011	Hospitalization	Total cost, procedure and surgery, anesthesia, surgical equipment, premium medical treatment, room and board, medication and injections, tests/radiology	10,818	6,485	–	8,870	958	–	–	–	–	–	–	–
Park et al. (24)	Colectomy	70 (35 LAP, 35 RAS)	September 2009 to July 2011	Hospitalization	Total cost, surgery and consumables, anesthesia, other therapy, consultation, laboratory, radiology, room charges, medical therapy	12235	10,320	–	–	–	–	8,714	5,110	–	3,159	5,209	–
Baek et al. (25)	Colorectal Surgery	304 (150 LAP, 154 RAS)	July 2007 to August 2010	Hospitalization	Total cost, operation, anesthesia, laboratory, radiology, nursing care, medical therapy, total hospital consumables, others	14647	9,978	–	8,849	2,289	–	11,540	3,956	–	3,107	6,022	–
Yoo et al. (38)	Thyroidectomy	211 (165 ENP, 46 RAS)	May 2009 to February 2011	Hospitalization	Operation cost	–		–	6,655	829	–	–	–	–	–	–	–
Yu et al. (26)	Liver surgery	30 (17 LAP, 13 RAS)	July 2007 to October 2011	Hospitalization	Total cost	11,475	6,762	–	–	–	–	–	–	–	–	–	–
Park et al. (27)	LAR^*^	217 (84LAP, 133 RAS)	April 2006 to August 2011	Hospitalization	Total cost	12,743	10,101	–	–	–	–	10,029	4,285	–	2,713	5,816	–
Kim et al. (28)	LAR	468 (234 LAP, 234 RAS)	January 2007 to December 2011	Hospitalization	Total cost, operation cost, anesthesia, preoperative diagnosis, postoperative management	15,965	11,933	–	10,375	6,796	–	12,613	5,104	–	3,352	6,829	–
Kim^†^ et al. (29)	Gastrectomy	434 (211 LAP, 223 RAS)	May 2011 to December 2012	Hospitalization	Total median cost (convert it to mean)	16,203	9,681	–	–	–	–	11,536	3,692		4,034	6,152	
Kang et al. (42)	Right hemicolectomy	96 (43 LAP, 20 RAS, 33 OPEN)	June 2007 to December 2011	Hospitalization	Total cost, Patients' out of pocket cost	12,492	9,911	9,009	–	–	–	8,788	4,394	3,487	3,704	5,517	5,522
Park et al. (30)	Right colectomy	70 patients (35 LAC and 35 RAC)	January 2010 to November 2011	Hospitalization	Operation cost	12,235	10,319	–	–	–	–	–	–	–	–	–	–
Yun^†^ et al. (43)	Prostatectomy	1,228 (170 LAP, 559 RAS, 135 OPEN)	January 2010 to December 2011	From admission to one year after discharge (hospitalization cost extracted)	Total cost, hospitalization, operation cost, post–op outpatients	18,312	7,414	4,775	14,253	4,073	1,599	15,826	2,784	1,437	–	–	–
Yoon et al. (31)	Choledochal cyst excision	39 (23 LAP, 16 RAS)	January 2005 to December 2018	Hospitalization	Total cost, operation, anesthesia, postoperative management	7331	6,568	–	5,781	4,810	–	6,578	2,626	–	753	3,942	–
Eoh et al. (44)	Hysterectomy	5,065 (3,248 LAP, 315 RAS, 1,503 OPEN)	January 2012 to December 2016	Post–discharge	Post–operative Emergency Room (ER) and outpatient (OP) visit cost	OP: 298 ER: 633	OP: 349 ER: 781	OP: 533 ER: 1,974	–	–	–	OP:15 ER:75	OP:18 ER:67	OP:75 ER: 136	OP: 283 ER: 558	OP: 330 ER: 715	OP: 506 ER: 1,838
Jang et al. (39)	Radical hysterectomy	62 patients (20 SP and 42 MP)	November 2011 to July 2017	Hospitalization	Total cost	SP: 5,655 MP: 8,190	–	–	–	–	–	–	–	–	–	–	–
Choi et al. (37)	Thyroidectomy	1033 patients (531 OT and 502 RT)	December 2018 to March 2020	Hospitalization	Operation cost	–	–	–	7,331	–	854	–	–	–	–	–	–
Cho et al. (32)	Cholecystectomy	100 patients (50 RC and 50 LC)	March 2017 to January 2019	Hospitalization	Total Cost	7,355	4,815	–			–	–	–	–	–	–	–
Han et al. (40)	Lobectomy	142 (53 RAS 2 ports, 89 RAS 3 ports)	January 2017 to April 2020	Hospitalization	Operation cost, Total medical cost	2ports: 16,806 3ports: 15,836	–	–	2ports: 11,080 3ports: 10,297		–	–	–	–	–	–	–
Choi et al. (33)	Adrenalectomy	56 (24 RA and 32 LA)	October 2018 ~ March 2022	Hospitalization	Operation cost	–	–	–	5,289	442	–	–	–	–	–	–	–
Shin et al. (34)	Distal Pancreatectomy	42 (21 RP and 21 LP)	January 2015 ~ September 2020	Hospitalization	Total Cost	15,722	12,699	–	–	–	–	–	–	–	–	–	–
Yoon^†^ et al. (35)	Hysterectomy	268 (173 LAP, 95 RAS)	March 2016 and May 2022	Hospitalization	Total cost, patient payment, government payment	12,123	6,884	–	–	–	–	9,155	2,567	–	2,881	4,184	–
Heo et al. (41)	Pyeloplasty	28 (14 RAS SP, 14 RAS MP^*^)	January 2010 and December 2020	Hospitalization	Total cost, operation cost, hospitalization cost	SP: 8,053 MP: 6,354	–	–	SP: 7,372 MP: 5,848	–	–	–	–	–	–	–	–
Hyun et al. (36)	Gynecologic surgery	367 patients (197 SPL and 170 SSR)	July 2020 to December 2023	Hospitalization	Out–of–pocket expenses paid by the patient at discharge	–	–	–	–	–	–	7,221	1,170	–	–	–	–

^*^LAR, Low Anterior Resection; SP, Single port; MP, Multi-port; OP, Outpatient visit; ER, Emergency room visit; ^†^reported median cost.

### 3.3 Risk of bias assessment

**Figure 3:**
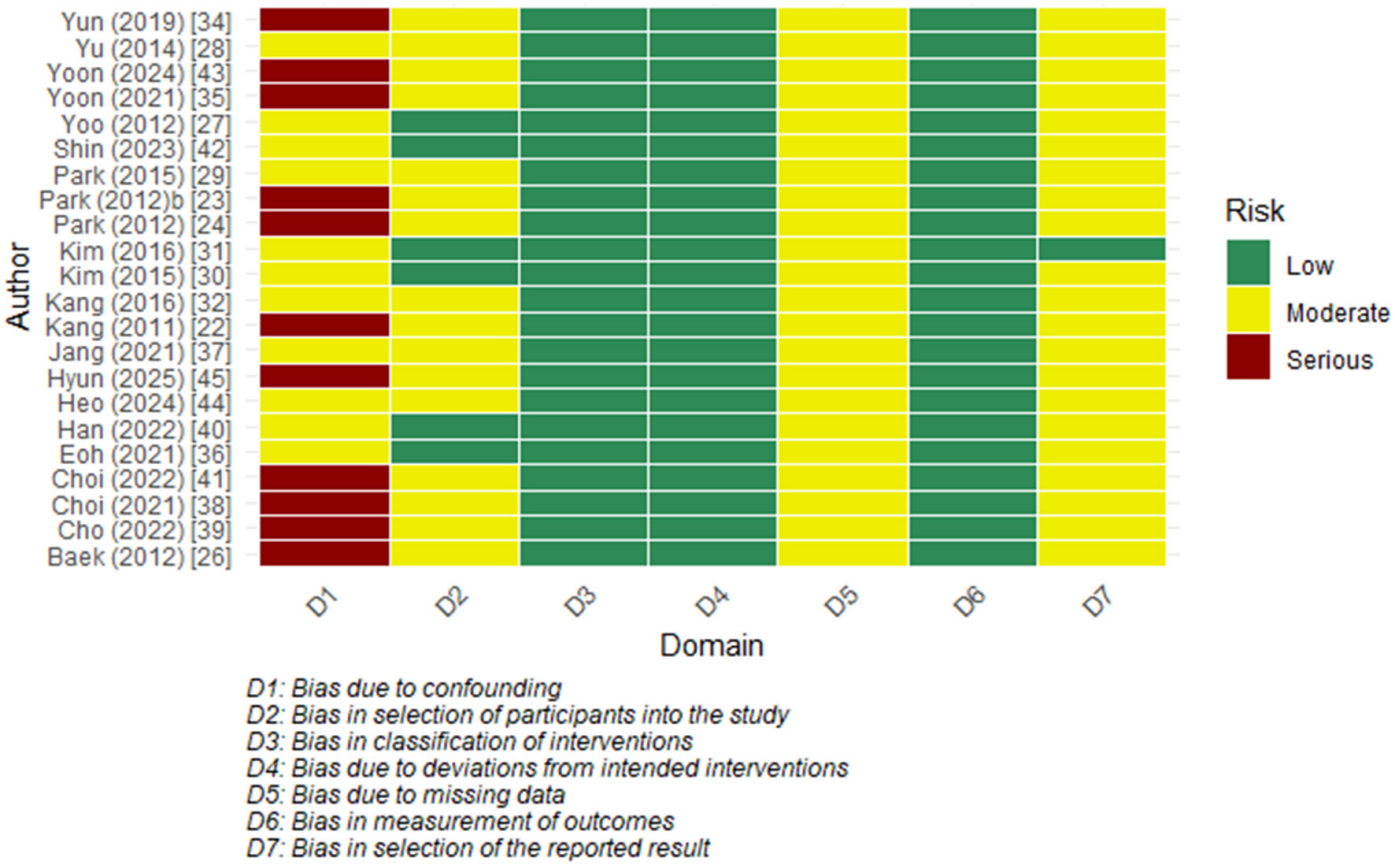
Risk of bias heatmap (ROBINS-I Domains).

The two RCTs were assessed as having a low risk of bias across all 5 domains (Table 2). Among the observational studies, the majority were found to have an overall moderate risk. The confounding domain (D1) often showed a ‘serious' level of bias, primarily due to a lack of statistical adjustment and the possibility of unmeasured factors. Other domains, including bias in measurement classification of interventions (D3) and bias in measurement of outcomes (D6), were mostly assessed as low risk. Most studies were rated as having a moderate risk in selecting the reported results (D7) as there were no prespecified protocols (Figure 3).

**Table 2:** Risk of bias (Randomized trials).

**References**	**D1**	**D2**	**D3**	**D4**	**D5**
Park et al. (24 )	Low	Low	Low	Low	Low
Park et al. (30)	Low	Low	Low	Low	Low

D1, Bias arising from the randomization process; D2, Bias due to deviations from intended interventions; D3, Bias due to missing outcome data; D4, Bias in measurement of the outcome; D5, Bias in selection of the reported result.

### 3.4 Surgical techniques and surgical specialties

All 24 publications included were comparative studies. Of these, 83 % (*N* = 20) were two-arm studies, 13% (*N* = 3) three-arm studies and 4% (*N* = 1) four-arm studies. Among the two arm studies, the majority (*N* = 15) compared RAS with laparoscopic surgery (22–36). One study compared RAS with open surgery (37), and another compared RAS with endoscopic surgery (37, 38). Three studies compared different RAS port configurations (39–41). Among three-arm studies, all three studies compared RAS, laparoscopic surgery, and open surgery (42–44). The four-arm study, which focused on radical nephrectomy, evaluated RAS, laparoscopic surgery, open surgery, and video-assisted mini laparotomy surgery (VAMS) (45).

### 3.5 Cost comparison by surgical specialties

Of the 24 studies, a total of 7 different surgical specialties were covered. The most frequently represented specialty was colorectal surgery, reported in six studies (24, 25, 27, 28, 30, 42), followed by Hepato-Biliary-Pancreatic (HBP) surgery in five studies (22, 26, 31, 32, 34) and gynecology in four studies (35, 36, 39, 44). Endocrine surgery (33, 37, 38) and urology were each represented in three studies (41, 43, 45), while gastrointestinal surgery appeared in two studies (23, 29). One study included thoracic surgery (40). Regarding surgical indications, 18 studies focused on cancer surgeries (23–30, 34, 35, 38, 40, 42–45), while four studies focused on benign diseases (31, 32, 36, 41). In addition, two studies included both benign and malignant indications. One study investigated adrenal glands (33), and the other on pancreatic lesions (22).

#### 3.5.1 Colorectal surgery

In total, six studies were identified in colorectal surgery either addressing rectal or colon cancer (24, 25, 27, 28, 30, 42). Five studies reported the total cost of care during hospitalization (24, 25, 27, 28, 42) while one study reported operation cost. Of the five studies reporting total hospitalization costs, two provided cost breakdowns that allowed identification of operation-related costs (25, 28). The mean total hospitalization cost ranged from $10,101 to $15,965 for RAS, $9,911 to $12,743 for laparoscopic surgery and $9,009 (reported in a single study) for open surgery. The operation costs ranged from $8,849 to $12,235 for RAS, while laparoscopic surgery ranged from $2,289 to $10,320. The out of pocket (OOP) payment ranged from $8,714 to $12,613 for RAS, $3,956 to $5,110 for laparoscopic, and $3,487 for open surgery, respectively.

#### 3.5.2 Hepato-biliary-pancreatic (HBP) surgery

Among the HBP surgery studies, two investigated pancreatectomy (22, 34), one examined cholecystectomy (32), one addressed choledochal cyst excision (31), and one involved liver resection (26). All studies reported total hospitalization costs covering the period from the admission to discharge. The reported total hospitalization costs for RAS ranged from $7,331 to $15,722, while laparoscopic surgery ranged from $4,815 to $12,699. In terms of operation cost and OOP payment, two studies provided data (22, 31). For pancreatectomy, the operation cost was $5,753 for RAS and $2,222 for laparoscopic surgery. Choledochal cyst excision was $5781 for RAS and $4,810 for laparoscopic surgery. The mean OOP for choledochal cyst excision was $6,578 for RAS and $2,626 for laparoscopic surgery.

#### 3.5.3 Gynecology

Hysterectomy was the dominant procedure among the gynecology publications. Two studies focused on endometrial cancer (35, 44), one addressed cervical cancer (39), and one evaluated benign gynecologic procedures (36). In one cervical cancer study, costs were compared between single-port robotic hysterectomy and multi-port radical hysterectomy (39). The single-port approach was associated with a cost of $5,655, compared to $8,190 for the multi-port procedure. One study on endometrial cancer compared total hospitalization costs from admission to discharge between RAS and laparoscopic surgery, reporting $12,123 for RAS and $6,884 for laparoscopy (35). The OOP payments were $9,155 for RAS patients and $2,567 for laparoscopic patients. Another endometrial cancer study analyzed post-discharge costs across RAS, laparoscopic, and open hysterectomy using a nationwide NHIS database (44). The post-discharge costs were $298 for RAS, $349 for laparoscopic, and $533 for open surgery. The study on benign gynecologic surgeries reported that OOP payments were $7,221 for RAS and $1,170 for laparoscopic surgery (36).

#### 3.5.4 Urology

Three publications were identified in urology (41, 43, 45). One study compared the single-port and multi-port approaches within RAS for pyeloplasty (41). The total hospitalization cost for single-port was $8,053 and $6,354 for multi-port. Another study examined radical nephrectomy for renal cancer patients (45). The mean total costs were $6,762 for RAS, $2,039 for laparoscopic surgery, and $1,706 for open surgery. The third study included both hospitalization cost and one year post discharge cost of radical prostatectomy, reporting the total median cost for each modality (43). The median total costs were $18,312 for RAS, $7,414 for laparoscopic surgery and $4,775 for open surgery. The operation costs were $14,253 for RAS, $4,073 for laparoscopic surgery and $1,599 for open surgery.

#### 3.5.5 Endocrine surgery

In endocrine surgery, there were two studies. The first compared robotic thyroidectomy (RT) to open thyroidectomy (OT). The second compared robotic thyroidectomy (RT) to endoscopic thyroidectomy (ET) (37, 38). Both studies reported only operation costs. The mean operation cost for robotic thyroidectomy was $6,655 to $7,331, for open thyroidectomy was $854, and for endoscopic thyroidectomy was $829. The mean operation cost of adrenalectomy was $5,288 for RAS and $442 for laparoscopic surgery (33).

#### 3.5.6 Gastrointestinal surgery

Two studies on gastrectomy were identified (23, 29). One study assessed gastrectomy and reported that the median total costs were $12,505 for RAS and $8,225 for laparoscopic surgery (29). The other study assessed distal gastrectomy for gastric cancer, showing that the mean total costs of distal gastrectomy were $10,818 for RAS and $6,485 for laparoscopic surgery (23).

#### 3.5.7 Thoracic surgery

A thoracic surgery study compared two-ports to three-ports in RAS surgery for lobectomy (40). The results indicated that the three-port was less costly than the two-port. The mean total costs were $16,806 for the two-ports lobectomy and $15,836 for the three-ports lobectomy. Similarly, the operation cost was $11,080 for two-ports and $10,297 for three-ports.

### 3.6 Meta-analyses of pooled outcomes between RAS and laparoscopic surgery

Pooled analyses of costs between two surgical modalities were performed using the studies comparing the economic outcomes between RAS and laparoscopic or endoscopic surgery during hospitalization. High heterogeneity was observed in the meta-analyses due to the inclusion of multiple types of surgical procedures, where clinical and economic characteristics were different.

#### 3.6.1 Total hospitalization cost

**Figure 4:**
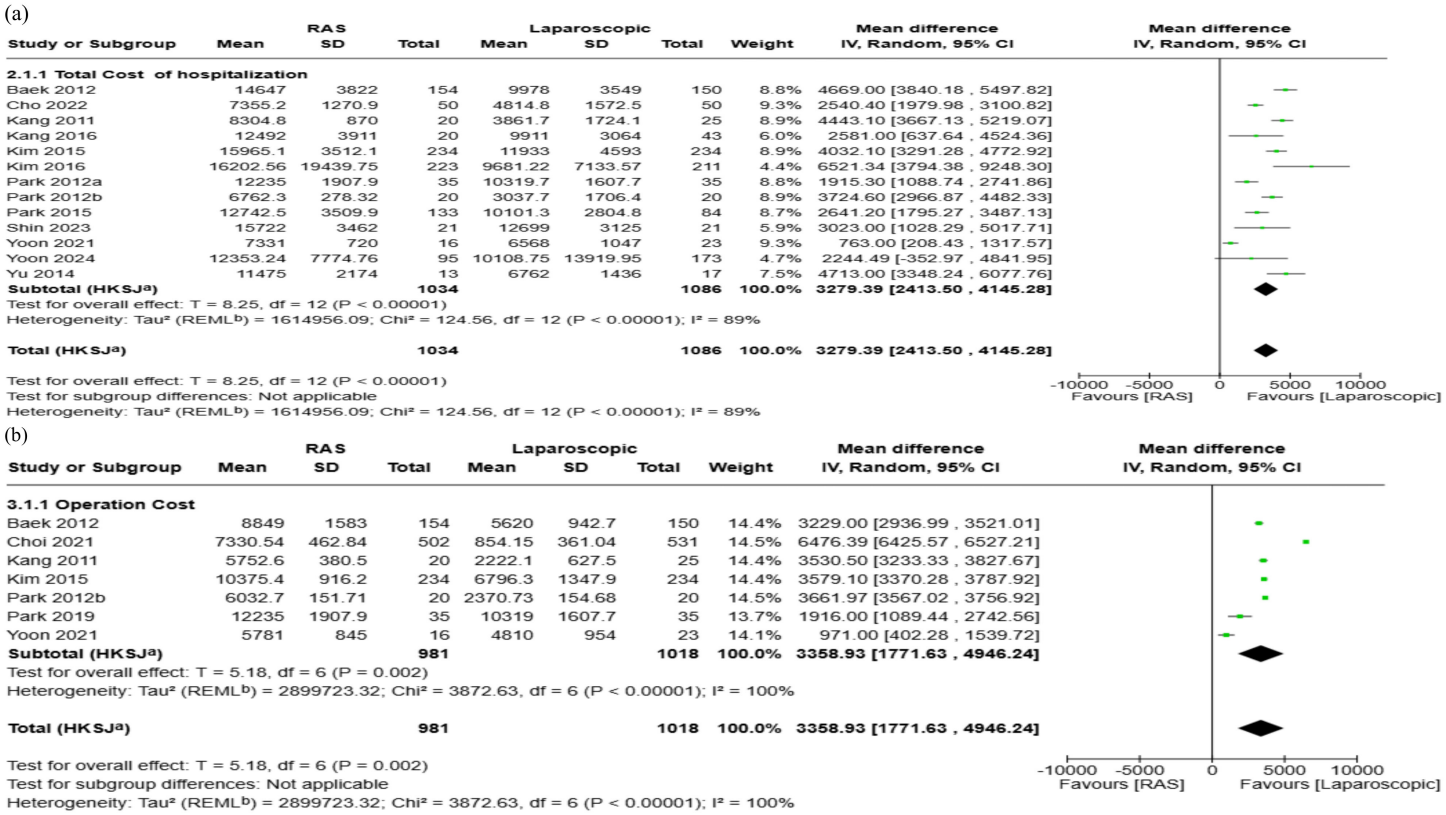
Forest plots comparing the costs of robot-assisted surgery (RAS) and laparoscopic/endoscopic/VATS surgery: **(a)** total hospitalization cost, and **(b)** operation cost.

**Figure 5:**
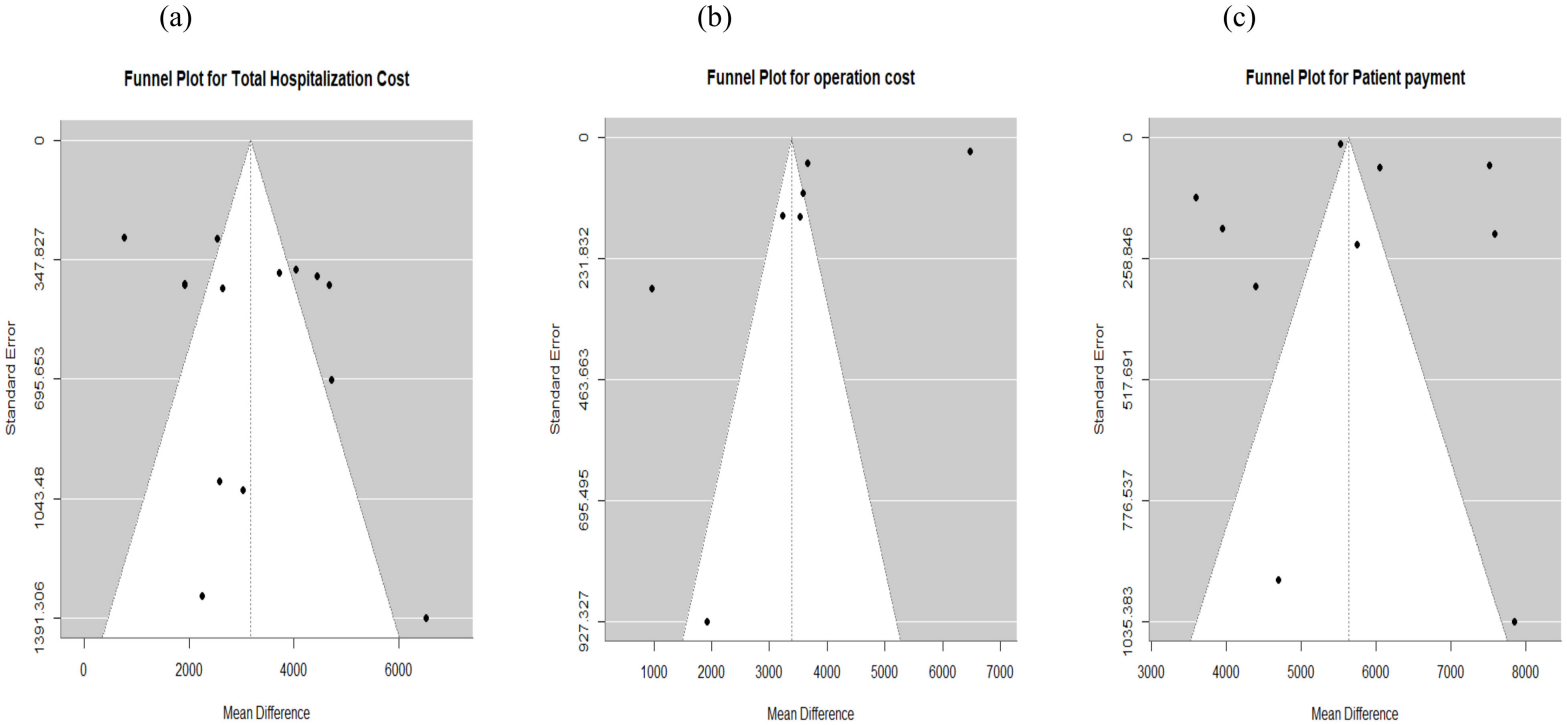
Funnel plot for publication bias assessment. **(a)** Total cost. **(b)** Operation cost. **(c)** Patient payment.

Fourteen studies reporting data for total hospitalization cost for RAS and laparoscopic were included in the meta-analysis. The pooled mean difference in total hospitalization cost from admission to discharge was $3,279 higher for RAS when compared with laparoscopic surgery (95% CI: $2,414 to $4,145; *I*^2^ = 89%) (Figure 4a). Visual assessment of the funnel plot and Egger's regression test (*p* = 0.3016) indicated that there was no significant publication bias (Figure 5a).

#### 3.6.2 Operation cost

For operating cost, 9 studies reported a total of 2,205 patients (RAS: 1,035 patients; laparoscopic: 1,170 patients). The pooled analysis showed that RAS was associated with higher mean operating costs, with a pooled mean difference of $3,359 (95% CI: $1,771 to $4,946; *I*^2^: 100%) (Figure 4b). Publication bias assessment using funnel plot and Egger's regression test (*p* = 0.18) showed that there is no significant publication bias, which was supported by visual inspection (Figure 5b).

#### 3.6.3 Patient payment (Out of pocket payment)

**Figure 6:**
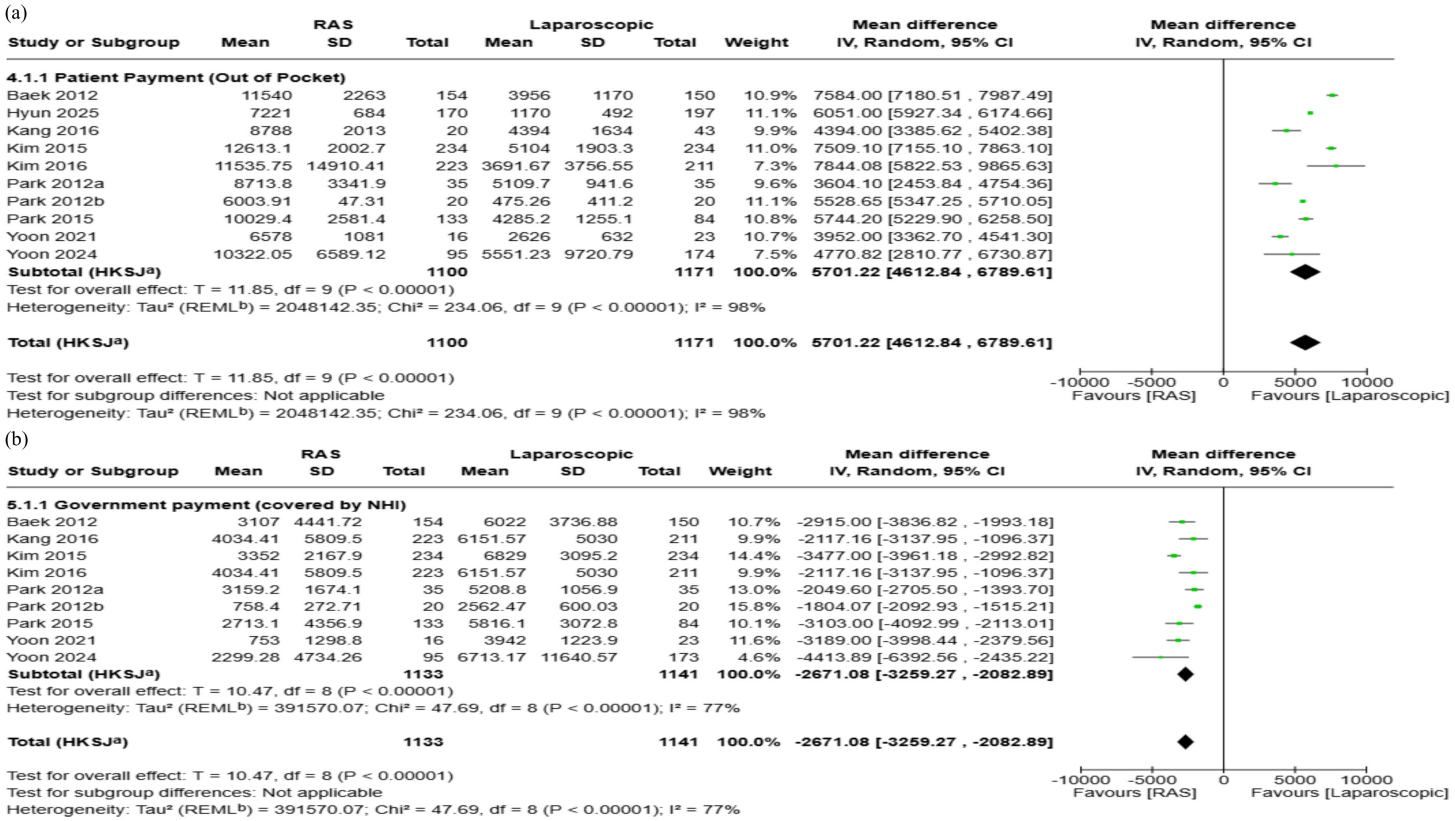
Forest plots comparing the costs of robot-assisted surgery (RAS) and laparoscopic/endoscopic/VATS surgery: **(a)** patient payment (out-of-pocket, OOP), and **(b)** government payment (covered by NHI).

Ten studies reported patients' payments during hospitalization. The pooled analysis showed that RAS incurred $5,701 higher patient costs compared with laparoscopic surgery (95% CI: $4,613 to $6,790; *I*^2^ = 98%) (Figure 6a). Publication bias was assessed using funnel plot and Egger's test, which indicated no publication bias in patient OOP (*p* = 0.79). Visual assessment of the funnel plot supported the absence of publication bias (Figure 5c).

#### 3.6.4 Government payment

Nine studies were included in this analysis. For studies that did not directly report government payments, values were estimated by subtracting OOP from total hospitalization cost. The pooled analysis showed that government payment was $2,671 lower in RAS compared to laparoscopic surgery (95% CI: $2,083 to $3,259; I^2^:77%) (Figure 6b). Publication bias was not evaluated for government payment as several summary statistics were estimated or imputed, potentially violating the assumptions required for interpreting funnel plot.

### 3.7 Sensitivity analyses

In sensitivity analyses excluding colorectal studies, the results were largely consistent with those of the overall analysis, showing no substantial difference from the colorectal group. When stratified by indication, cost differences between RAS and laparoscopic surgery were minimal in malignant conditions, which were predominantly colorectal, but were more pronounced in benign conditions. However, the number of studies addressing benign indications was very limited, and these findings should therefore be interpreted with caution (Supplementary 3).

## 4 Discussion

This is the first and most comprehensive systematic literature review of economic studies on RAS, dedicated solely to the South Korean setting. Across all cases, RAS was associated with higher overall costs compared with alternative surgical modalities. The reported hospitalization cost for RAS ranged from $6,762 to $20,206, compared with $3,038 to $11,933 for laparoscopic or endoscopic surgery and $1,706 to $9,009 for open surgery. Depending on the procedure and study, the total hospitalization costs of RAS were 1.1 to 2.2 times higher than those of laparoscopic or endoscopic surgery, and 1.4 to 4.0 times higher than for open surgery.

Given that RAS is not covered by national health insurance, the patient cost burden is substantial. Currently, patients in Korea are paying the full cost of RAS procedures, meaning that access to innovative surgical technology is largely determined by one's ability to pay. Throughout the studies, patient payments for RAS were 1.71 to 12.63 times greater than those for other surgical modalities. However, government payments for RAS procedures were only 19% to 67% of those for laparoscopic or endoscopic surgeries. Many countries reported that the extent of access to advanced technologies often varies by insurance status, leading to further disparities in health outcomes (46–48). In Korea, Eoh et al. (44) reported that individuals with lower income or from non-metropolitan region were more likely to receive open and laparoscopic surgery than RAS (44). Our findings also demonstrate that patients' financial burden for RAS is considerably higher than that of other surgical modalities.

To reduce disparities in access to new technologies based on patients' insurance coverage or income status, the need for public reimbursement for RAS has been long discussed in Korea. However, one of the key challenges in advancing this discussion is the lack of robust evidence on the cost-effectiveness of RAS from a Korean perspective. In that sense, this review also highlights the limited availability of relevant cost-effectiveness data. While RAS is associated with high initial hospitalization costs, some studies in our review provided detailed breakdown of cost components (23–25, 28, 31, 43, 45), showing that certain cost categories—such as inpatient-wards, diagnostic tests, medical supplies and medications—were lower in the RAS group. These findings suggest that RAS may generate savings in specific areas, potentially due to shorter recovery times or better post-operative outcomes.

Previous systematic literature reviews have reported that adopting a societal perspective, using a longer time horizon, and analyzing higher surgical volumes tend to produce more favorable cost-effectiveness conclusions for RAS (49–51). For example, Sadri et al. (49) found that 81% of prostatectomy studies concluded that RAS was more cost-effective when modeling approaches were applied. Similarly, Song et al. (50) found that 91% of cost-effectiveness studies favored RAS, with 11 of 12 studies being long-term cost-utility analyses incorporating quality-adjusted life years (QALYs). These international findings highlight the importance of long-term cost-effectiveness studies that assess how improved post-operative outcomes may translate into long-term cost savings. In contrast, among the 24 studies in our review, only two studies reported post-discharge costs (43, 44). None of the studies included full economic evaluations with quality-of-life outcomes assessed over an extended time horizon. Consequently, the current evidence from Korea does not allow firm conclusions.

Beyond these differences in cost-effectiveness evidence, another important distinction between Korea and international studies lies in study design and data sources. While international analyses often draw upon multicenter or nationwide datasets, most Korean studies were single-institution case series, limiting the generalizability of their findings. These gaps underscore the need for more comprehensive, long-term, and multicenter evaluations in Korea to generate policy-relevant evidence on the cost-effectiveness of RAS.

However, there had been various challenges that made conducting such studies difficult. One major barrier to conducting a comprehensive cost-effectiveness study in Korea is the limited usability of the national claims database for evaluating non-reimbursed technologies such as RAS. Multiple studies attempted to identify RAS procedures in the national claims database using an operational definition (2, 44), but issues with accuracy and validation remain. Another challenge lies in the distinct reimbursement and pricing structure of RAS vs. laparoscopic surgery. RAS is billed as a bundled charge determined by the hospital, while laparoscopic or open surgeries are publicly reimbursed based on a fee-for-service model that includes both reimbursed and non-reimbursed components. The lack of transparency in itemized components used during surgery makes it difficult to generate cost estimates for open and laparoscopic procedures that are directly comparable to the bundled charges for RAS.

To the best of our knowledge, this study marks the first attempt to comprehensively map the economic literature on RAS in the Korean setting. Through this review, we identified the current status of existing economic literature, key evidence gaps including the lack of long-term cost-effectiveness studies and modeling-based analyses, and areas that warrant greater academic attention. Nevertheless, this study has several limitations. First, to estimate the overall cost difference between RAS and other modalities, we pooled cost data across multiple specialties. The pooled estimates did not account for the considerable cost differences observed between surgeries for malignant and benign indications. Many of the reviewed studies were colorectal studies, potentially biasing the overall results toward the cost patterns observed in this specific specialty. In addition, the very high heterogeneity observed in our pooled estimates (*I*^2^ = 89–100%) reflects the diversity of surgical procedures, patient populations, and institutional settings represented across the included studies. This level of heterogeneity limits the interpretability of the pooled estimates and suggests that the results should be viewed as broad indications of overall cost differences rather than precise effect sizes. Second, 85% of the cost data was collected before 2020, with the earliest record from 2005, limiting our ability to provide up-to-date insights, particularly for the past 5 years. In Korea, the costs of open and laparoscopic surgeries have increased over the past 10 years. For example, the resource based relative value scale (RBRVS) of prostatectomy increased by 138% from 6,339.37 in 2015 to 15,081.24 in 2025. The third revision of the RBRVS in 2024, which included payments for surgery and laparoscopic consumables, may also influence the relative cost gap between RAS and conventional approaches. In this context, a more recent study encompassing the latest changes needs to be conducted. In addition, policy insights emphasize the importance of incorporating such up-to-date analyses to better inform future health policy and resource allocation

Ultimately, as new technologies are increasingly adopted across multiple surgical specialties, high-quality economic evaluations should accompany clinical innovations. Such evaluations are essential to guide sustainable and equitable healthcare development and resource allocation. In a country where RAS is not covered by national health insurance, the lack of robust economic evidence poses a critical challenge to inform decision making in coverage decisions. As the use of RAS expands, it is essential to address the current lack of comprehensive and methodologically rigorous economic studies through improved data availability and research quality. Future research should aim to establish a stronger and more comprehensive economic evidence base by incorporating QALYs and adopting a societal perspective. The development and wider adoption of alternative data sources such as surgical registries and Common Data Models (CDMs) will further facilitate robust economic evaluation (52). To ensure equitable access to advanced healthcare technologies and improve health outcomes, stronger collaboration among research communities, policymakers, and healthcare providers, alongside sustained political commitment, will be essential.

## 5 Conclusion

This is the first systematic review and meta-analysis to evaluate the economic implications of RAS in Korea. Our findings indicate that RAS is associated with higher costs compared with other surgical approaches, yet robust evidence of its long-term cost-effectiveness remains insufficient. As the adoption of RAS continues to expand, generating high-quality real-world data will be essential to inform equitable and evidence-based reimbursement decisions.

## Data Availability

The original contributions presented in the study are included in the article/Supplementary material, further inquiries can be directed to the corresponding author.
