# Supplementary material for: Costs and cost-effectiveness of robotic-assisted surgery in South Korea: a systematic review and meta-analysis
Source: Front Public Health. 2025 Oct 17;13:1683482. doi: 10.3389/fpubh.2025.1683482 (PMC12576917; doi:10.3389/fpubh.2025.1683482)
Supplement: Supplementary file 2 [file Table_2.docx]

**Supplementary Material 2. Search Strategy used in database**

| Database | Search terms | Results |
| --- | --- | --- |
| Pubmed | (cost* OR economic* OR financial* OR pric* OR charge* OR billing*)  AND  Korea  AND  ('robot surgery' OR 'robot assisted*' OR 'robot-assisted*' OR 'robotic surgery' OR 'robotic-assisted*')  Language: English; Publication date: 2005/1/1– | 237 |
| Embase | (“cost* OR economic* OR financial* OR pric* OR charge* OR billing*) AND (Korea) AND ("robot surgery" OR "robot-assisted*" OR "robotic surgery" OR "robotic-assisted*")”  Language: English; Publication date: 2005/1/1– | 322 |
| Scopus | (TITLE-ABS-KEY(cost* OR economic* OR financial* OR pric* OR charge* OR billing*)) AND (TITLE-ABS-KEY(korea)) AND (TITLE-ABS-KEY('robot surgery' OR 'robot assisted*' OR 'robot-assisted*' OR 'robotic surgery' OR 'robotic-assisted*'))  Publication date: 2005/1/1 - | 34 |

*Date of last search: May 8, 2025*
