# Supplementary material for: Costs and cost-effectiveness of robotic-assisted surgery in South Korea: a systematic review and meta-analysis
Source: Front Public Health. 2025 Oct 17;13:1683482. doi: 10.3389/fpubh.2025.1683482 (PMC12576917; doi:10.3389/fpubh.2025.1683482)

**Supplementary 3. Sensitivity Analyses**

**3-1. Pooled Estimates Excluding Colorectal Cases**

1. Total cost


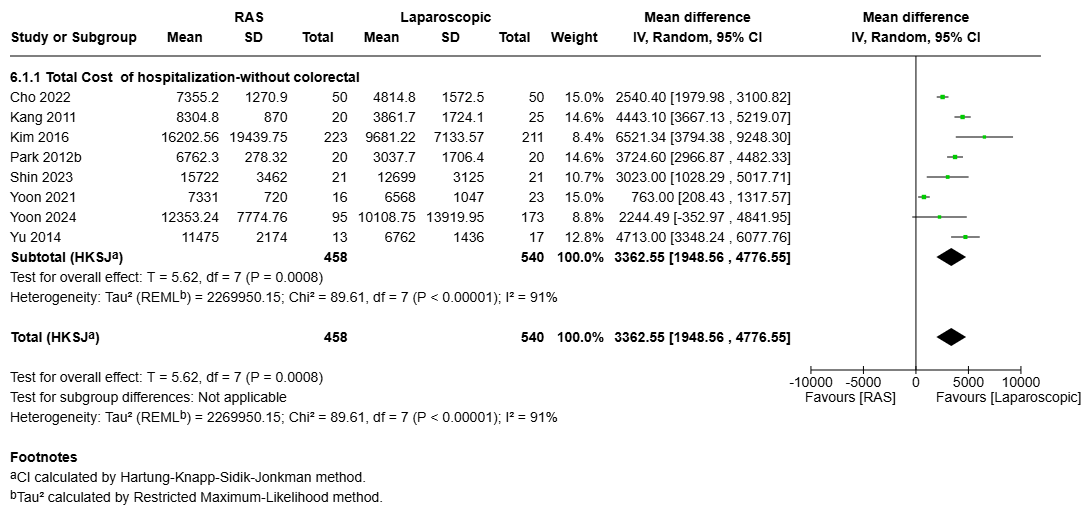


1. Operation cost


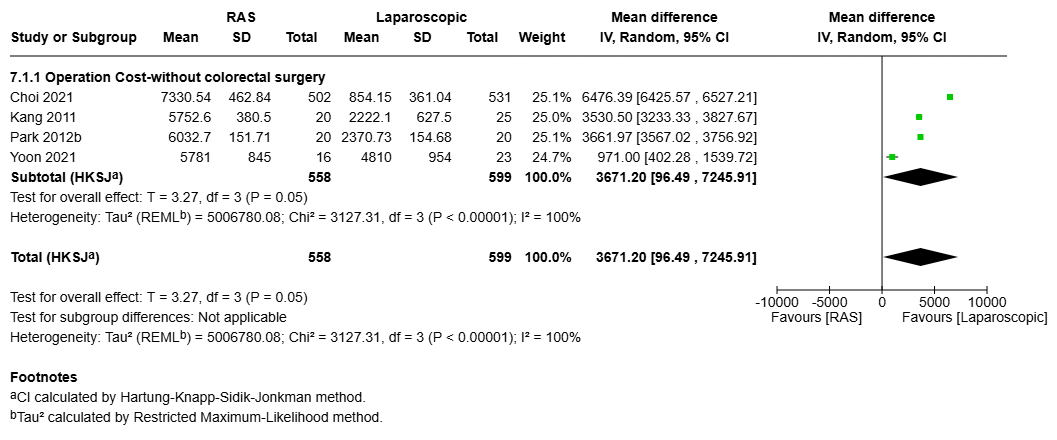


1. Out of pocket payment


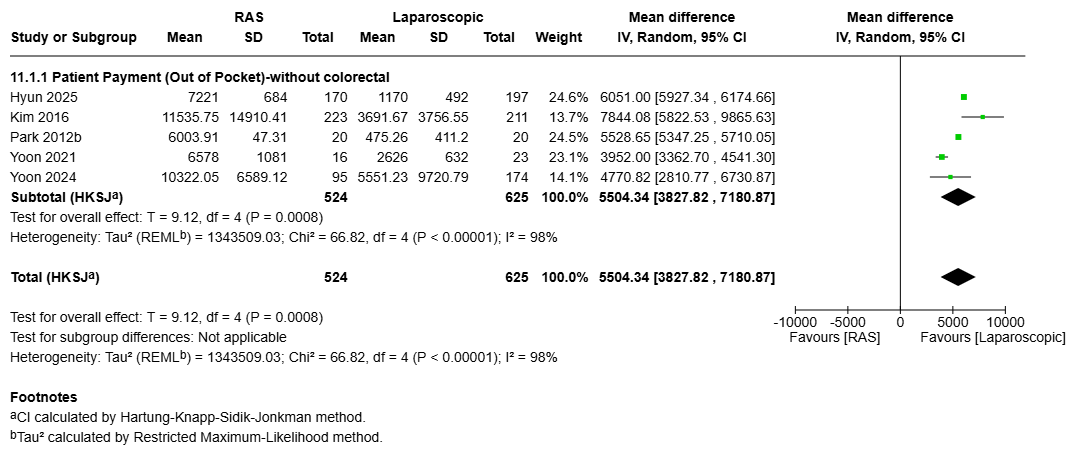


1. Government payment


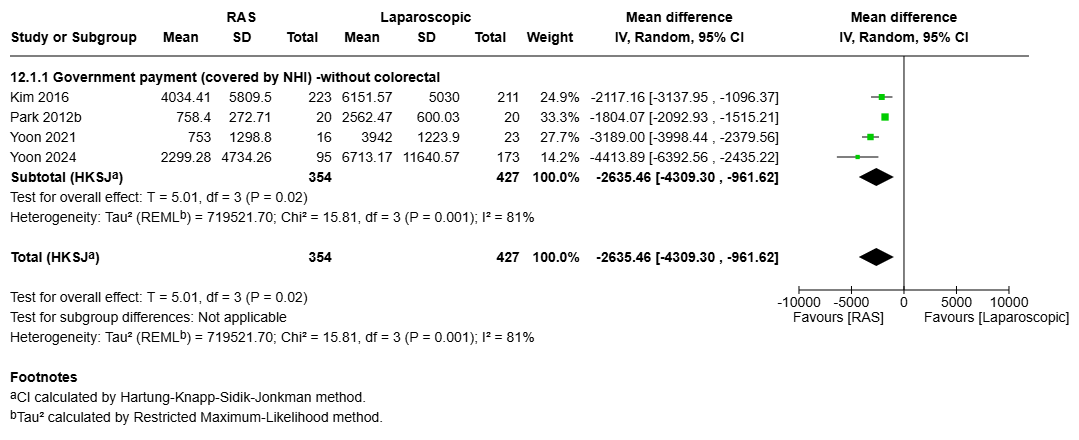


**3-1. Pooled estimates for malignant diseases**

1. Total cost


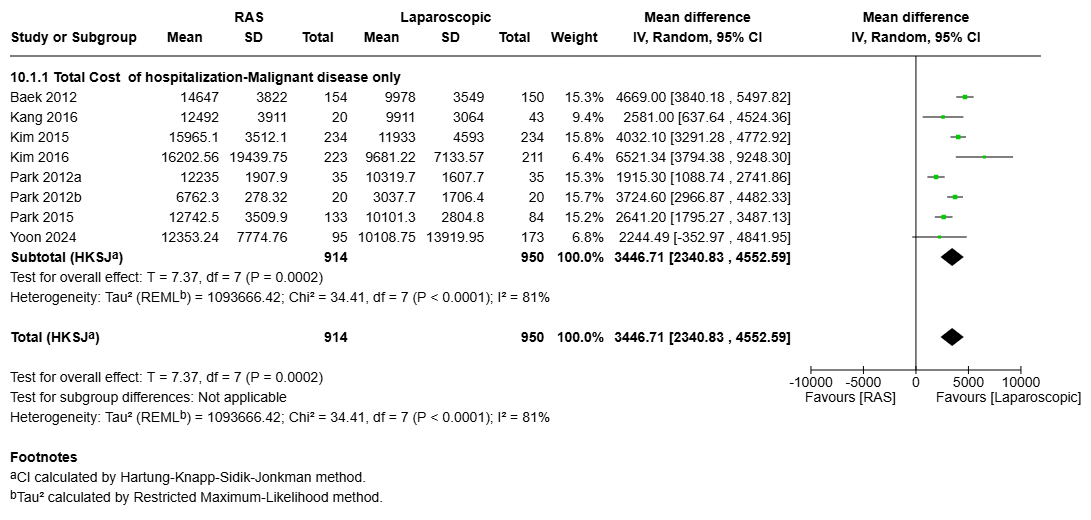


1. Operation cost


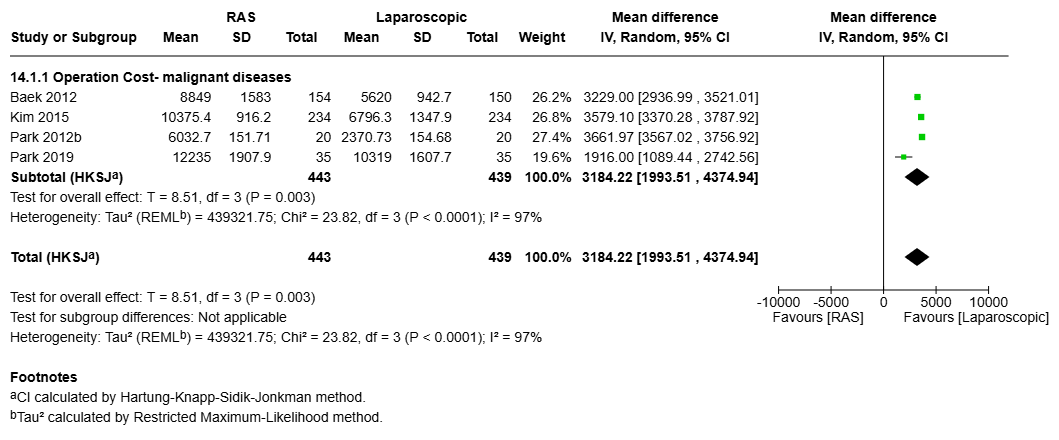


1. Out of pocket payment


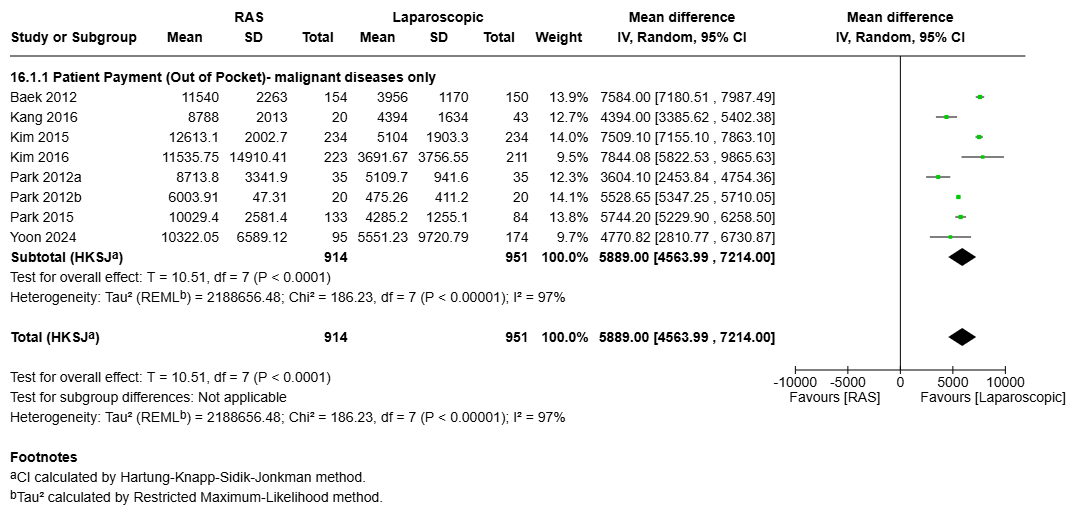


1. Government payment


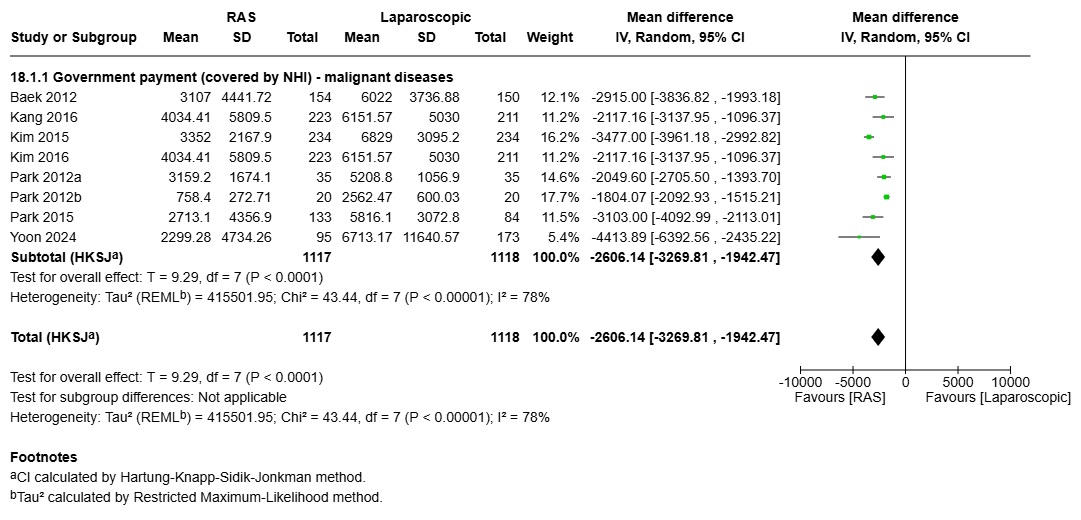


**3-3. Pooled estimates for benign diseases**

1. Total cost


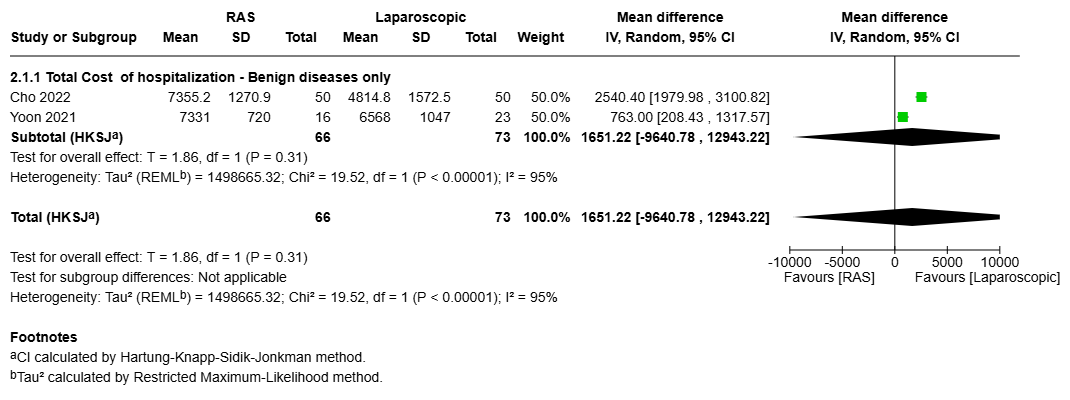


1. Operational cost


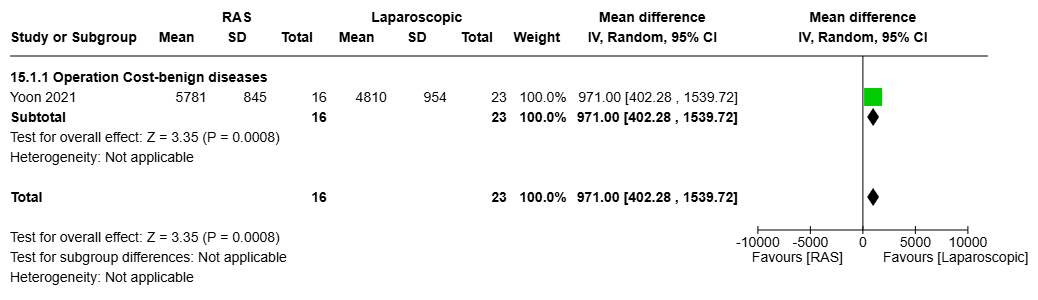


1. Out of pocket payment


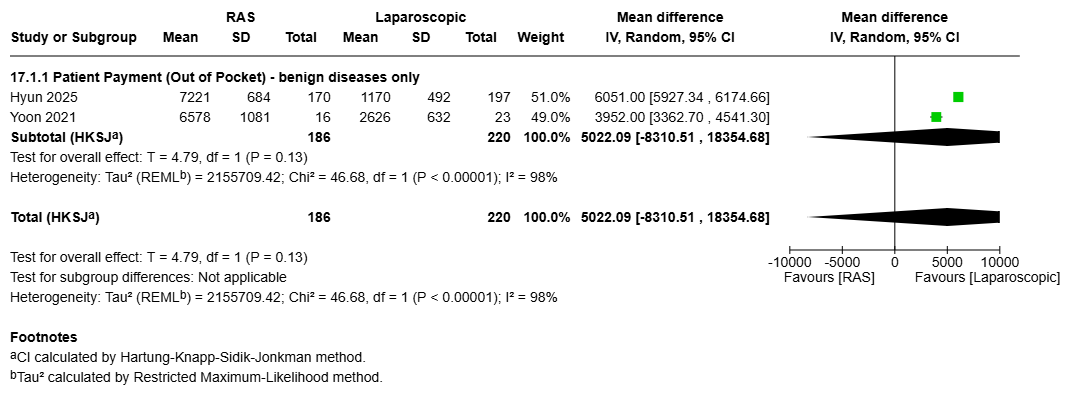


1. Government payment


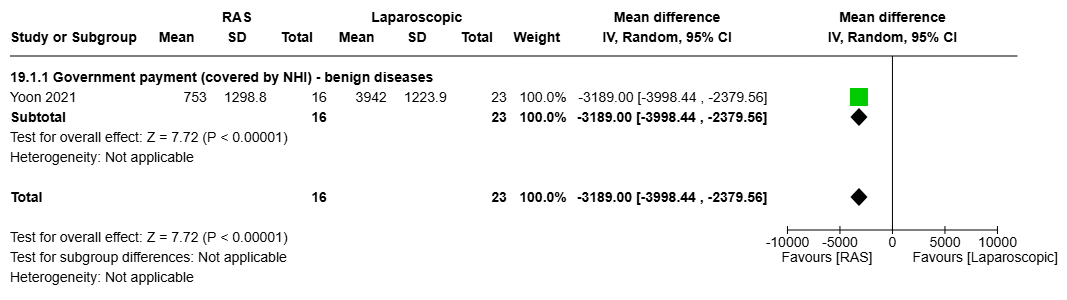

Supplement: Supplementary file 3 [file Table_3.docx]
